# Supplementary material for: Double-strand breaks in facultative heterochromatin require specific movements and chromatin changes for efficient repair
Source: Nat Commun. 2024 Oct 17;15:8984. doi: 10.1038/s41467-024-53313-2 (PMC11487122; doi:10.1038/s41467-024-53313-2)
Supplement: Supplementary file 1 — Supplementary Information [file 41467_2024_53313_MOESM1_ESM.pdf]

Supplementary information related to:

Double-strand breaks in facultative heterochromatin require specific movements and chromatin changes for efficient repair.

Marieke R. Wensveen[1], Aditya A. Dixit[1], Robin van Schendel[2], Apfrida Kendek[1], Jan Paul Lambooij[1], Marcel Tijsterman[2], Serafin U. Colmenares[3] and Aniek Janssen[1,*]

1) Center for Molecular Medicine, University Medical Center Utrecht, Universiteitsweg 100, 3584 CG, Utrecht, the Netherlands
2) Human Genetics Department, Leiden University Medical Center, Leiden, the Netherlands
3) Department of Molecular and Cell Biology, University of California Berkeley, Berkeley, USA
* Correspondence to: a.janssen-2@umcutrecht.nl

Content:
Supplementary Figure 1-8
Supplementary Table 1-2

SUPPLEMENTARY FIGURE 1

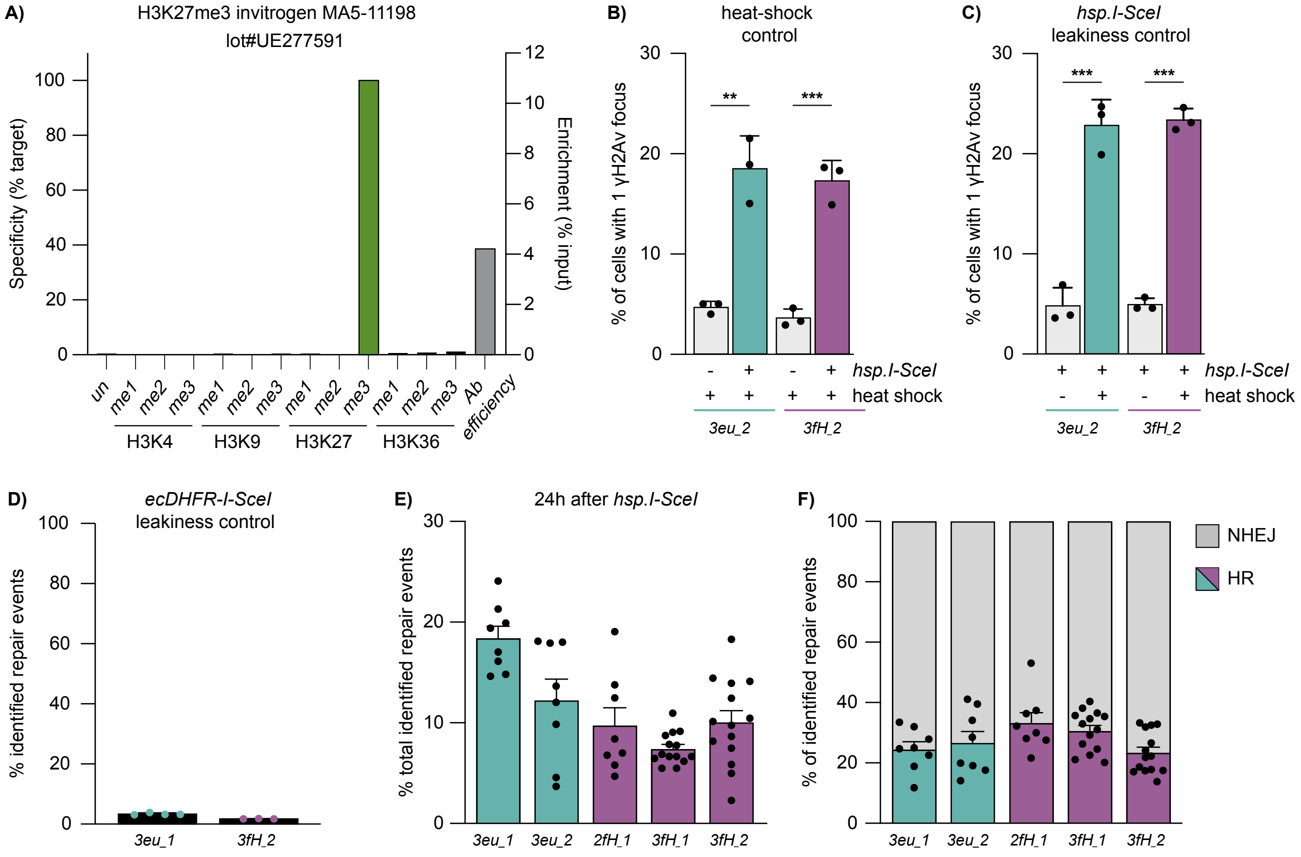

Supplementary Figure 1 (extension Fig.1). DR-*white* system to induce single DSBs in euchromatin and facultative heterochromatin.
A) ChIP specificity and efficiency of H3K27me3 antibody determined using EpiCypher K-MetStat panel. Bars indicate the specificity of the antibody (left axis) for the expected target modification (green) and off-target modifications (black). Grey bars (right y-axis) indicate the percentage of target modification pulldown relative to input (measure of antibody efficiency). **B, C**) DR-*white* larvae were heat-shocked at 37C for one hour. Number of DSBs per cell was quantified in the presence and absence of the *hsp.I-SceI* transgene (**B**) as well as before and 6 hours after heat-shock in the presence of *hsp.I-SceI* (**C**) using immunofluorescence staining for γH2Av (to visualize DSBs) and DAPI (to visualize nuclei). Bars indicate averages +SD of 3 wing discs per DR-*white* site (n≥160 cells per wing disc). **D**) DR-*white/ecDHFR-I-SceI* larvae were grown on standard food (no trimethoprim). DR-*white* reporters were subjected to PCR, Sanger sequencing and analyzed for repair products using the TIDE algorithm. Graph shows quantification of total identified repair events. **E, F**) DR-*white/hsp.I-SceI* larvae were heat-shocked for one hour to induce I-SceI. Repair products were subjected to PCR and Sanger sequencing 24 hours after I-SceI induction and analyzed using the TIDE algorithm[37]. Graphs show quantification of total identified repair events (**E**) and the percentage of homologous recombinatino (HR, color) and non-homologous end joining (NHEJ, grey) repair products (**F**). Bars indicate averages +SEM of 8 independent experiments (larvae) per condition (except 3fH_1 and 3fH_2: n=14). If not shown, p-value not significant (>0.05), (**) p-value≤0.01, (***) p-value≤0.001, two-sided unpaired t-test (**B, C**), one-way ANOVA + Tukey's multiple comparisons test (**F**). Source data and exact p-values are provided as Source Data file.

SUPPLEMENTARY FIGURE 2

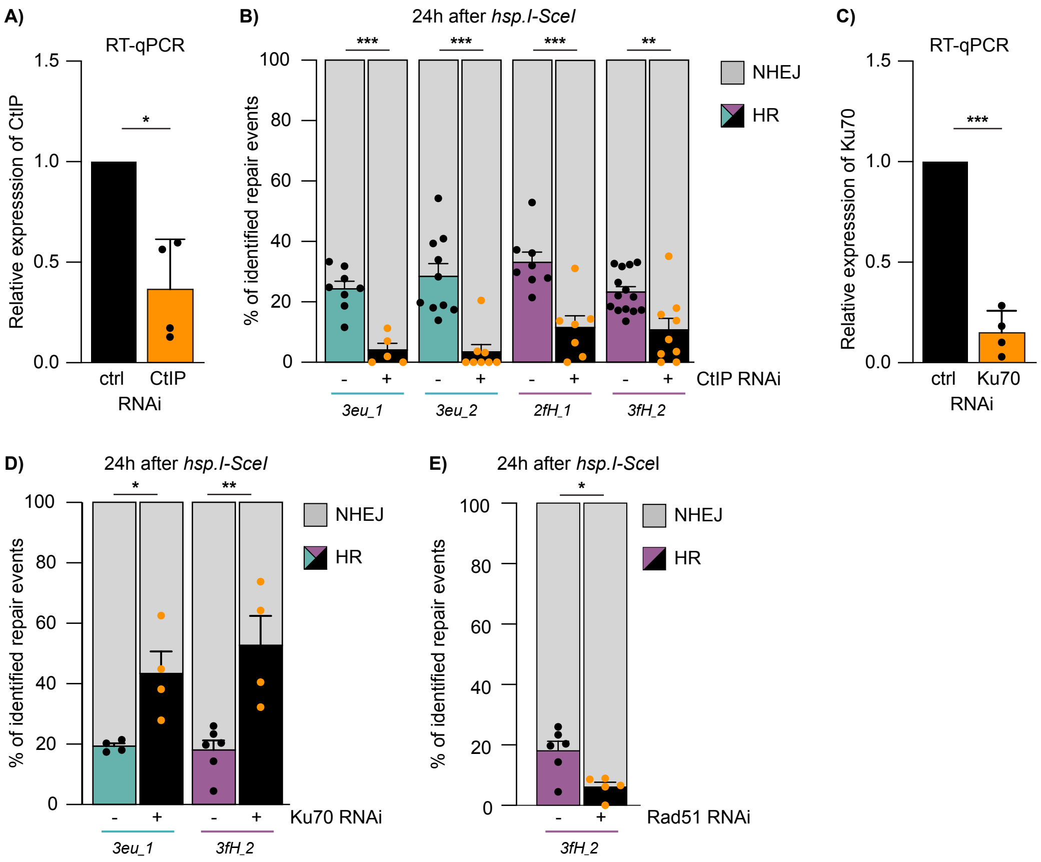

Supplementary Figure 2 (extension Fig.1). DR-*white* system to induce single DSBs in euchromatin and facultative heterochromatin.
A, C) Relative *DmCtIP* (**A**) and *DmKu70* (**C**) expression level in 3rd instar larvae normalized to an internal control gene (*vermillion*), determined using Reverse Transcription followed by quantitative PCR (RT-qPCR). Bars indicate averages +SD of 4 single larvae per condition (luciferase control RNAi, DmCtIP RNAi or DmKu70 RNAi). **B, D, E**) DR-*white/hsp.I-SceI* larvae with indicated dsRNA expression were heat-shocked for one hour to induce I-SceI. 24 hours after I-SceI induction repair products were analyzed using the TIDE algorithm[37]. Graphs show quantification of the percentage homologous recombination (HR, magenta/purple/black) and non-homologous end joining (NHEJ, grey) repair products. Bars indicate averages +SEM of ≥4 independent experiments (larvae) per condition. (*) p-value≤0.05, (**) p-value≤0.01, (***) p-value≤0.001, two-sided paired t-test (**A, C**) and two-sided unpaired t-test (**B, D, E**). Source data and exact p-values are provided as Source Data file.

SUPPLEMENTARY FIGURE 3

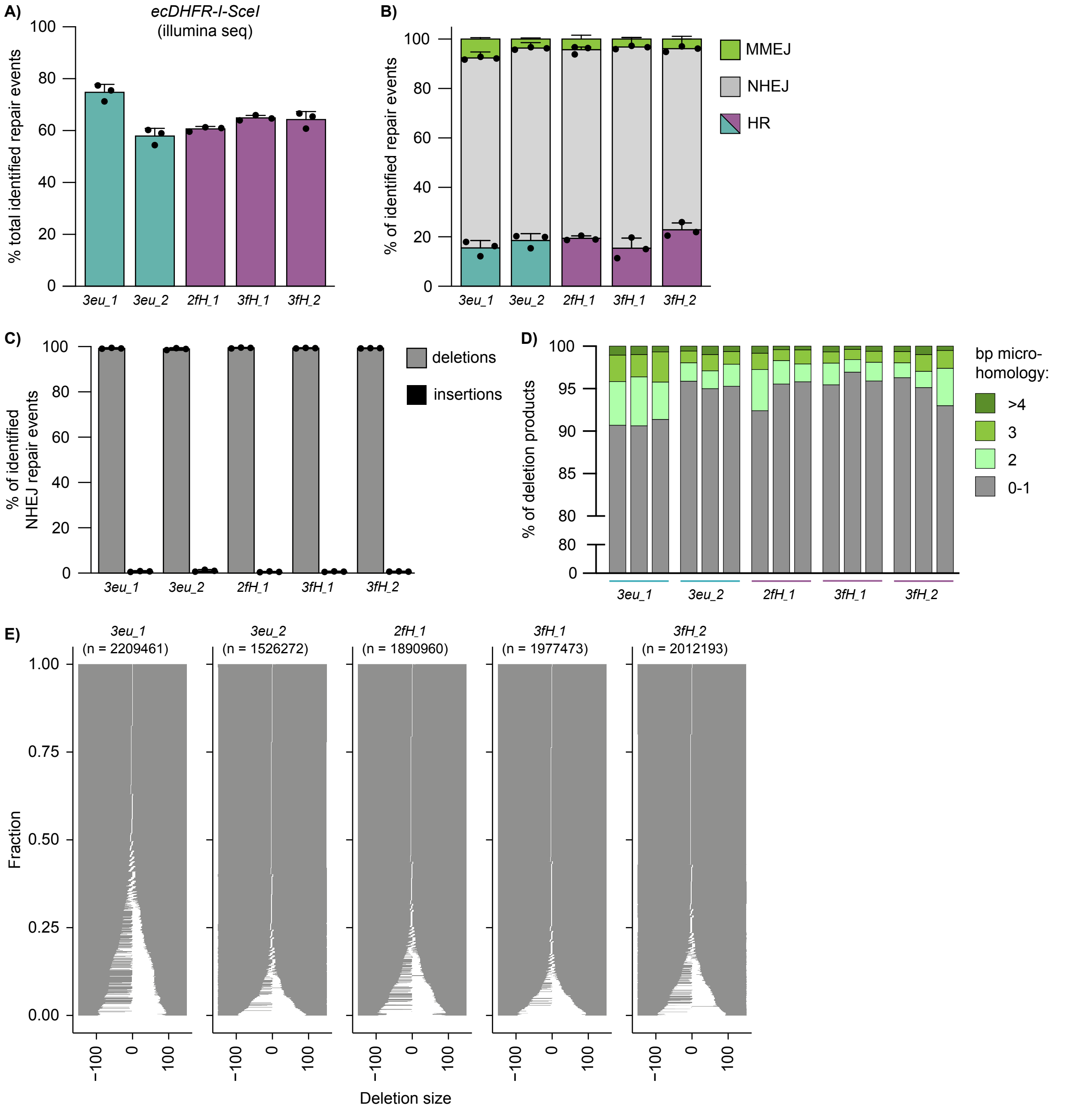

Supplementary Figure 3 (extension Fig.1). DSB repair product analysis in euchromatin and facultative heterochromatin using Illumina sequencing. A-E) DR-*white/ecDHFR-I-SceI* larvae were fed trimethoprim for 3-4 days to induce I-SceI. Repair products at the upstream *white* gene were PCR amplified, Illumina sequenced and analyzed using the SIQ algorithm[63]. Graphs show quantification of total identified repair events (**A**) and the percentage of repair products with either homologous recombination (HR, magenta/purple), non-homologous end joining (NHEJ, grey) or microhomology mediated alternative end-joining (MMEJ, green) signatures (**B**). Averages are shown +SD of 3 independent experiments (larvae) per condition. **C**) Quantification of the percentage of NHEJ products with deletions (grey) or insertions (black) identified by Illumina sequencing of the indicated DR-*white* insertions. Bars indicate average percentages +SD of 3 independent experiments (larvae) per condition. **D**) Quantification of the percentage of deletion products with 0-1, 2, 3 and 4 or more basepair microhomology identified by Illumina sequencing at indicated DR-*white* integration sites. Individual samples (larvae) are displayed. **E**) SIQPlotteR tornado plot visualization of the deletion profile at indicated DR-*white* integrations. Data is plotted relative to the I-SceI break site (at 0) and sorted by deletion size. The white space represents the deletion size for each event. Source data are provided as Source Data file.

SUPPLEMENTARY FIGURE 4

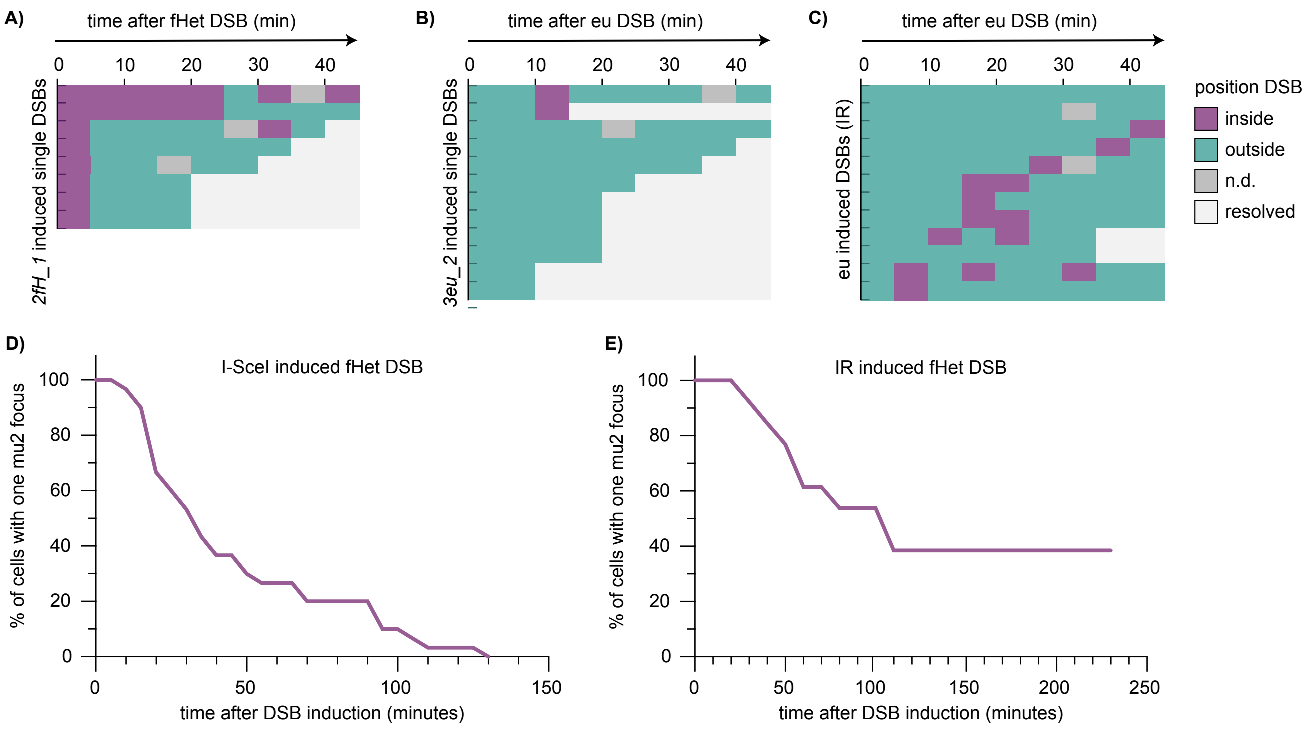

Supplementary Figure 4 (extension Fig.2). Facultative heterochromatic DSBs move outside polycomb bodies.
A-C) Quantification of live imaging of Mu2-eYFP (DSB) dynamics and kinetics relative to ph-p-mCherry domains. Rows indicate single Mu2-eYFP foci from the timepoint they appear (0 min) within (2fH_1 + *ecDHFR-I-SceI* (**A**)) or outside (3eu_2 + *ecDHFR-I-SceI* (**B**), 5Gy gamma-radiation (IR) (**C**)) polycomb bodies until the timepoint they resolve. Mu2-eYFP foci were followed up to 45 minutes (min) after their appearance. Colors indicate localization of the Mu2-eYFP focus with respect to ph-p domains (inside polycomb body [purple], outside [green], not detectable [grey], or resolved [white]). **D, E**) Time-lapse analysis of ecDHFR-I-SceI induced mu2-eYFP foci (**D**) and IR induced mu2-eYFP foci (**E**) in facultative heterochromatin. Disappearance (minutes from appearance) in third instar wing discs is plotted. The time point of mu2-focus appearance was set at t=0 for each individual focus. Source data are provided as Source Data file.

SUPPLEMENTARY FIGURE 5

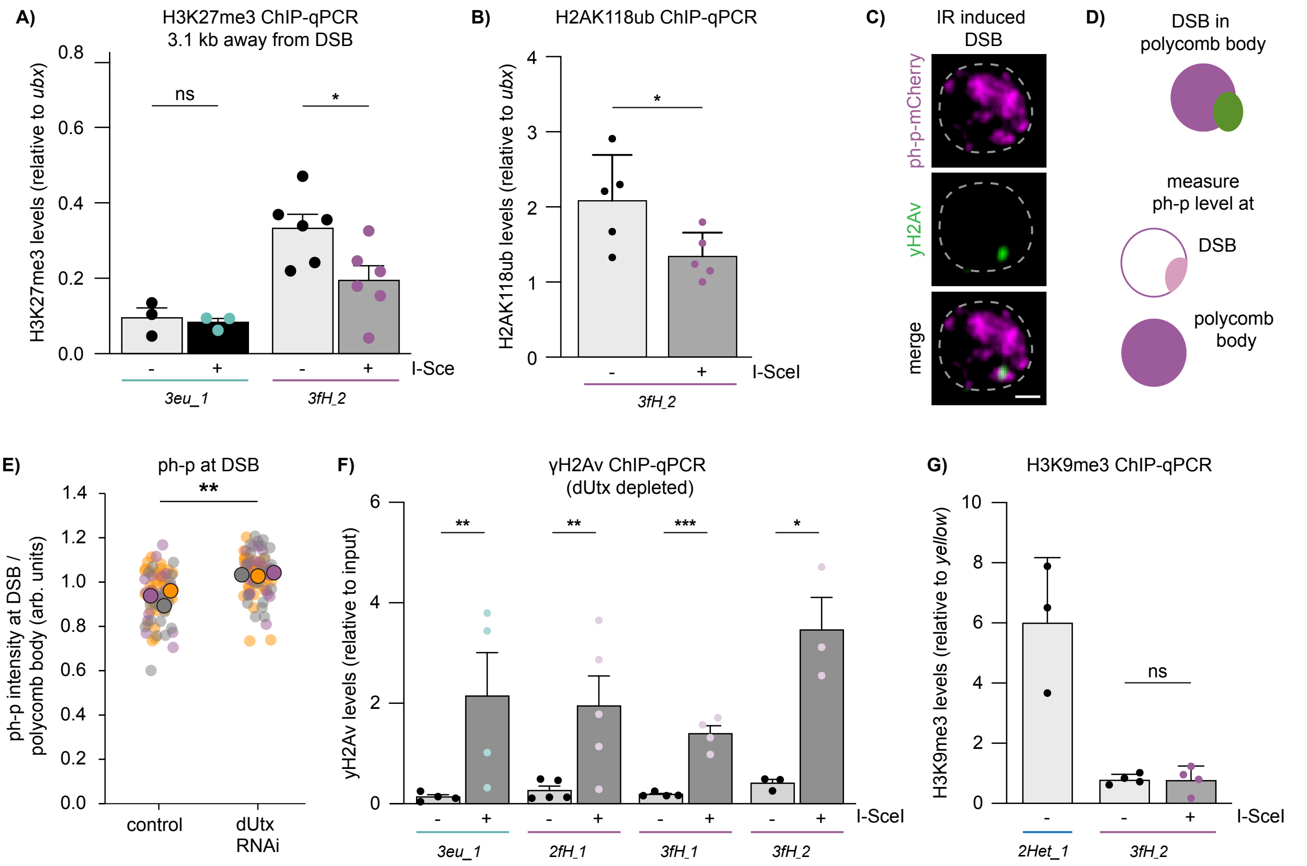

Supplementary Figure 5 (extension Fig.3). DSBs in facultative heterochromatin lose fHet chromatin marks.
A, B) ChIP-qPCR analysis for H3K27me3 (**A**) and H2AK118Ub (**B**) in the absence or presence of a DSB (-/+ hsp.I-SceI) in indicated conditions (as in Fig 1F, using qPCR primers 3.1 kb away from the DSB (A) or regular qPCR primers 1.4 kb away from the DSB (**B**)). H2AK118Ub and H3K27me3 levels were normalized using a ubx qPCR primer set as internal control. Averages are shown for ≥3 for independent experiments +SD. **C**) Representative image of irradiation-induced yH2Av focus (DSB marker, green) within the ph-p domain (polycomb marker, magenta) in Kc cells within 30 minutes after 5Gy IR. Scale bar = 1μm. Dotted line outlines nucleus. **D**) Schematic of the quantification of ph-p levels at DSBs in polycomb bodies. Relative enrichment of ph-p at DSBs is calculated by dividing ph-p levels at the DSB (light pink) by ph-p levels at the complete polycomb body (magenta). **E**) Quantification of relative enrichment of ph-p levels at DSBs in polycomb bodies with or without dUtx depletion, within 30 minutes after IR (quantification as in Fig.S5D). Graph represents three independent experiments per condition. Each big circle (grey, purple, orange) represents the average intensity within one experiment. Small circles represent individual cells within one experiment, arb. units = arbitrary units. **F, G**) ChIP-qPCR analysis for yH2Av (**F**) and H3K9me3 (**G**) in the absence or presence of a DSB (-/+ hsp.I-SceI) in indicated conditions (as in Fig 1F). H3K9me3 levels were normalized using a yellow qPCR primer set as internal control. As a positive control, a (uncut) DR-white locus in constitutive heterochromatin is shown (2het_1)[22]. Averages are shown for ≥3 for independent experiments +SEM (**D**) or +SD (**E**). (ns) not significant, (*) p-value≤0.05, (**) p-value ≤0.01, two-tailed paired t-test (**B, G**), two-tailed unpaired t-test (**A, C**), ratio paired t-test (**F**). Source Data and exact p-values are provided as Source Data file.

SUPPLEMENTARY FIGURE 6

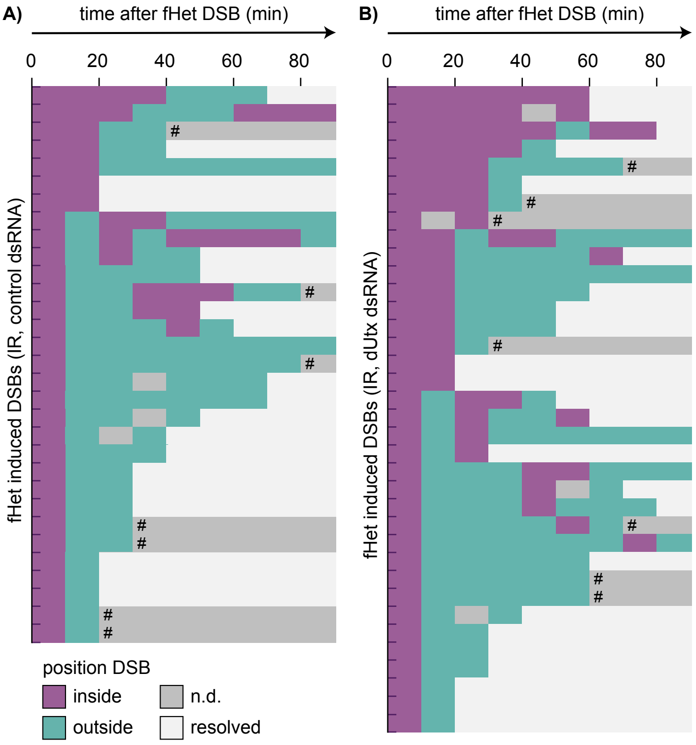

Supplementary Figure 6 (extension Fig.4). dUtx promotes DSB movement.
A, B) Quantification of live imaging of ATRIP-eYFP (DSB) dynamics and kinetics relative to the ph-p-mCherry domains upon 5Gy gamma-radiation (IR) to induce DSBs in control (**A**) or dUtx dsRNA expressing (**B**) Kc cells. Each row indicates one single Mu2-eYFP focus that appeared within a polycomb body. Mu2-eYFP foci were followed up to 90 minutes (min) after their appearance. Colors indicate localization of the Mu2-eYFP focus with respect to ph-p domains (inside polycomb body [purple], outside [green], not detectable [grey], or resolved [white]). # indicates DSBs that did not resolve during the course of our imaging experiment.

SUPPLEMENTARY FIGURE 7

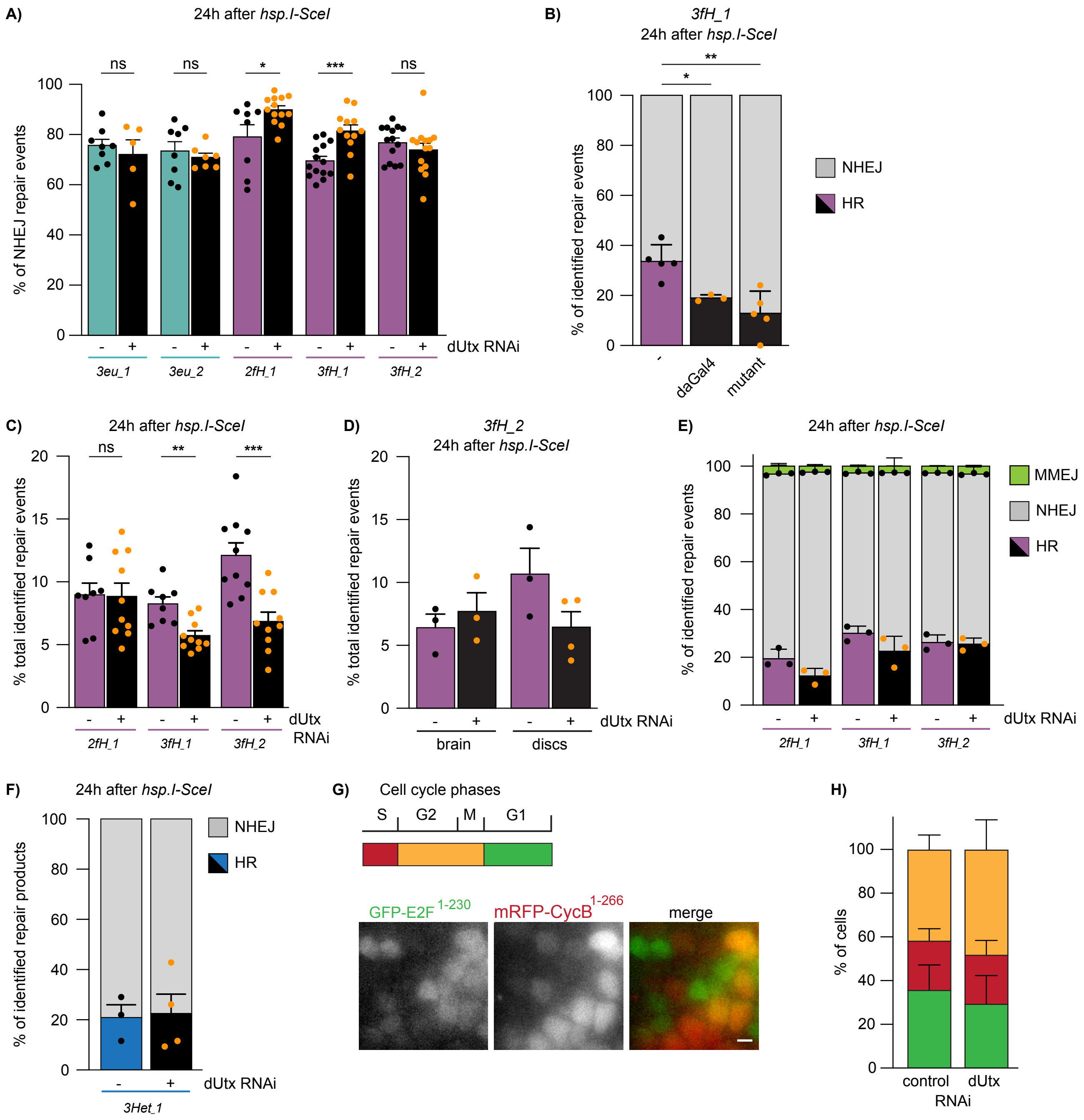

Supplementary Figure 7 (extension Fig.5). dUtx is important for HR at facultative heterochromatin DSBs.
A, C, D, F) DR-*white*/*hsp.I-SceI* larvae with (+) or without (-) dUtx RNAi were heat-shocked for one hour to induce I-SceI. Repair products of whole larvae (**A, C, F**) or indicated tissues (**D**) were Sanger sequenced 24 hours after I-SceI induction and analyzed using the TIDE algorithm[37]. Graphs show the percentage of identified non-homologous end joining (NHEJ) repair products (**A**), the percentage of repair products with either homologous recombination (HR) and NHEJ **(F)** and the quantification of total identified repair events (**C, D**). **B**) DR-*white*/*hsp.I-SceI* with dUtx RNAi driven by daughterless-Gal4 (*daGal4*), *dUtx*[f01321] mutant (mutant) or wildtype (-) larvae were used. **E**) DR-*white*/*hsp.I-SceI* repair products of the upstream *white* gene were PCR amplified, Illumina sequenced and analyzed using the SIQ algorithm. Graphs show the percentage of repair products with either HR (magenta/purple), NHEJ (grey) or microhomology mediated alternative end-joining (MMEJ, green) signatures. **F**) DR-*white* integration in constitutive heterochromatin (3het_1)[22] was used. **G**) Top: Schematic of distinct colors indicating each cell cycle phase in the Fly-FUCCI system. Bottom: Representative images of the Fly-FUCCI system in wing discs. Error bar = 2µm. **H**) Cell cycle analysis in wing discs of control (luciferase RNAi) and dUtx depleted (dUtx RNAi) larvae using the Fly-FUCCI system as illustrated in G. Colors indicate cell cycle phase (G1 [green], S [red], G2 and M [yellow]). Bars indicate averages +SD of at least 3 independent experiments (larvae) per condition (**A-F, H**). For B, one datapoint was removed, because it was assigned as an outlier according to the Grubbs test. If not shown, p-value not significant (>0.05), (*) p-value≤0.05, (**) p-value≤0.01, (***) p-value≤0.001, two-tailed unpaired t-test (**A-C, F, H**). Source data and exact p-values are provided as Source Data file.

SUPPLEMENTARY FIGURE 8

A)

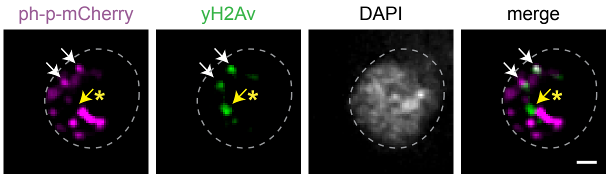

B)

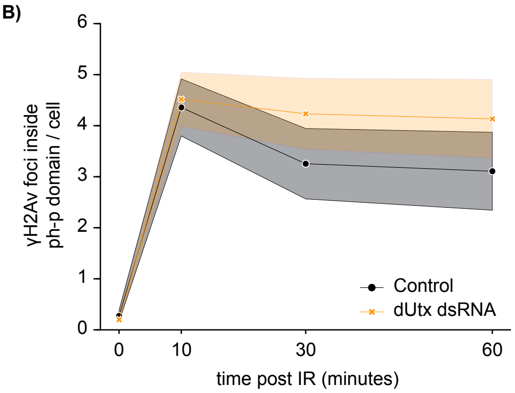

C)

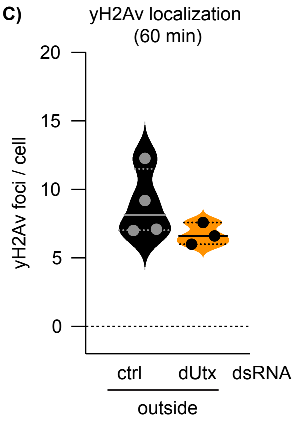

D)

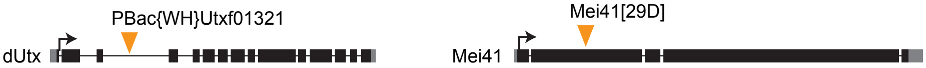

Survival	
Genotype	Viability
Mei41[29D]; dUtx+	1.0
Mei41+; dUtx[f01321]/CyO	0.6162
Mei41[29D]; dUtx[f01321]/CyO	0.5163
Relative viability of double mutant: 84%<br>p-value: 0.0001	

Survival after y-irradiation	
Genotype	Viability
Mei41[29D] ; dUtx+	0.1383
Mei41+; dUtx[f01321]/CyO	0.3185
Mei41[29D]; dUtx[f01321]/CyO	0.0190
Relative viability of double mutant: 6%<br>p-value: 0.0017	

Supplementary Figure 8 (extension Fig.5). dUtx promotes DSB movement and is essential for survival in an ATR-mutant background.
A) Representative image of a nucleus 60 minutes after 5Gy irradiation immunostained for anti-yH2Av (yellow), anti-Rad51 (cyan) and anti-mCherry to visualize ph-p-mCherry (magenta). White arrows indicate DSBs localizing 'inside' a polycomb body (the php-mCherry domain), yellow arrow (with star) indicates a DSB localizing 'outside' a polycomb body. Scale bar = 1µm. Dotted line outlines nucleus. **B**) Quantification of the number of yH2Av foci localizing inside the php-mCherry domain per cell in the absence (control, yellow dsRNA, black line) and presence of dUtx dsRNA (orange line). Average number of yH2Av foci inside the php-mCh domain per cell is shown at indicated time points. Shade represents SEM of 3 independent experiments (with ≥10 cells per condition). **C**) Quantification of the localization of yH2Av 60 minutes after 5Gy IR 'outside' a polycomb body. Dots represents ≥3 independent experiments per condition (with ≥10 cells per condition). Straight line in the violin plot indicates the median and dotted lines indicate quartiles. If not shown, p-value not significant (>0.05), two-tailed unpaired t-test (**B, C**). **D**) Synthetic lethality assay of dUtx heterozygous mutant with ATR (*mei41*) mutant in the absence (left) and presence (right) of 10 Gy irradiation. Offspring of five to 15 crosses were quantified. Relative viability of double mutant (*mei41[29D]*; *dUtx[f01321]*/*CyO*) was calculated relative to flies mutant for dUtx and wild type for ATR (*mei41+*; *dUtx [f01321]*/*CyO*). Source data and exact p-values are provided as Source Data file.

Supplementary Table 1. Fly lines.

**Fly lines**	**Origin**
ecDHFR-I-Sce	Janssen laboratory (Janssen et al., 2016)
hsp70.HA.I-Sce (hsp.I-Sce)	Jan LaRocque (Do et al., 2014)
mu2-eYFP	Karpen laboratory (Janssen et al., 2016)
php-mCh	created in this study
FlyFUCCI	BDSC #55123
UAS:dUtx RNAi	VDRC 105986
UAS:DmCtIP RNAi	VDRC 100035
UAS:DmKu70 RNAi	BDSC #29594
UAS:DmRad51 RNAi	VDRC 13362
UAS:luciferase RNAi	BDSC #31603
dUtx [f01321]	BDSC #18425
mei41[29D]	gift from Tin Tin Su
Act5C-Gal4	BDSC #4414 and #3954
Da-Gal4	BDSC #95282

**DR-white fly lines**	**Integration site**	**MiMIC line**	**BDSC #**
3eu_1	described in Janssen et al., 2016	MI03854	36965
3eu_2	3R (87A1) - 11876313	MI00256	33599
2fH_1	2R (60E3) - 24843209	MI02312	58609
3fH_1	3R (84A6) - 6866410	MI06379	60795
3fH_2	3R (90B5) -17590058	MI02651	36022
2Het_1	described in Janssen et al., 2016	MI00127	30628
3Het_1	described in Janssen et al., 2016	MI03233	36419

Supplementary Table 2. Primers.

qPCR primers	Goal	Fw	Rv
3xp3	1.4 kb away from I-SceI recognition site	CTCGCCCGGGGATCTAATTC	GCGACGTGTTCACTTTGCTT
tub	3.1 kb away from I-SceI recognition site	TGACTATGGTAGGTCGATATAA	AAGTTATACTAGAGAGCTTCG
yellow	internal control H3, yH2Av	ACGGTCCACAGAAGAGGATT	GCACTTAGCTCTAAGCTGACA
ubx	internal control H3K27me3	ACAGAGGATTCCCTCTCTCG	CAGGCAGTCCTGTTTGTAGG
CtIP	examine CtIP KD efficiency (larvae)	GAACTTGAAGCAAACTCGCC	CTTGACTGTGCTATTGCTG
vermilion	control for CtIP KD efficiency (larvae)	CTGCTCATGGACATCGACTC	GTGGACAGATTGAACAGATC
dUtx	examine dUtx KD efficiency (larvae + Kc cells)	CGGGGAGTTCCATATTGCCC	GGTACATCCATCCTAATTGTCGA
tubulin	control for CtIP KD efficiency (larvae)	GTGAAACACTTCCAATAAAAACTCAATATG	GCTCCAGTCTCGCTGAAGAA

Primers	Goal	Fw	Rv
DR-white	PCR primers repair product analysis	GGCCAGGGAACACCTGATTT	CGCGAATTCGTCGACATAAC
DR-white sequencing	sequencing primer repair product analysis	GAGCCCACCTCCGGACTGGAC	-
dsRNA dUtx	induce dUtx KD in Kc cells	TAATACGACTCACTATAGgcaacttcaacgccatgcc	TAATACGACTCACTATAGGgcgcattcacttgcaaaatgg
dsRNA yellow	induce control KD in Kc cells	TAATACGACTCACTATAGggaaaaactaagccaacgtcatc	TAATACGACTCACTATAGGgccgtggatataggcaaaaa

Primers for NGS	Sequence
NGS_P501_Fw	AATGATACGGCGACCACCGAGATCTACACTAGATCGCTCGTCGGCAGCGTCAGATGTGTATAAGAGACAGGACTGGACTCATTTACCGCCC
NGS_P502_Fw	AATGATACGGCGACCACCGAGATCTACACCTCTCTATTCGTCGGCAGCGTCAGATGTGTATAAGAGACAGGACTGGACTCATTTACCGCCC
NGS_P503_Fw	AATGATACGGCGACCACCGAGATCTACACTATCCTCTTCGTCGGCAGCGTCAGATGTGTATAAGAGACAGGACTGGACTCATTTACCGCCC
NGS_P504_Fw	AATGATACGGCGACCACCGAGATCTACACAGAGTAGATCGTCGGCAGCGTCAGATGTGTATAAGAGACAGGACTGGACTCATTTACCGCCC
NGS_P505_Fw	AATGATACGGCGACCACCGAGATCTACACGTAAGGAGTCGTCGGCAGCGTCAGATGTGTATAAGAGACAGGACTGGACTCATTTACCGCCC
NGS_P506_Fw	AATGATACGGCGACCACCGAGATCTACACACTGCATATCGTCGGCAGCGTCAGATGTGTATAAGAGACAGGACTGGACTCATTTACCGCCC
NGS_P507_Fw	AATGATACGGCGACCACCGAGATCTACACAAGGAGTATCGTCGGCAGCGTCAGATGTGTATAAGAGACAGGACTGGACTCATTTACCGCCC
NGS_P701_Rv	CAAGCAGAAGACGGCATACGAGATTCGCCTTAGTCTCGTGGGCTCGGAGATGTGTATAAGAGACAGTTGGTAGGACACTGGGCAC
NGS_P702_Rv	CAAGCAGAAGACGGCATACGAGATCTAGTACGGTCTCGTGGGCTCGGAGATGTGTATAAGAGACAGTTGGTAGGACACTGGGCAC
NGS_P703_Rv	CAAGCAGAAGACGGCATACGAGATTTCTGCCTGTCTCGTGGGCTCGGAGATGTGTATAAGAGACAGTTGGTAGGACACTGGGCAC
NGS_P704_Rv	CAAGCAGAAGACGGCATACGAGATGCTCAGGAGTCTCGTGGGCTCGGAGATGTGTATAAGAGACAGTTGGTAGGACACTGGGCAC
NGS_P705_Rv	CAAGCAGAAGACGGCATACGAGATAGGAGTCCGTCTCGTGGGCTCGGAGATGTGTATAAGAGACAGTTGGTAGGACACTGGGCAC
NGS_P706_Rv	CAAGCAGAAGACGGCATACGAGATCATGCCTAGTCTCGTGGGCTCGGAGATGTGTATAAGAGACAGTTGGTAGGACACTGGGCAC
NGS_P707_Rv	CAAGCAGAAGACGGCATACGAGATGTAGAGAGGTCTCGTGGGCTCGGAGATGTGTATAAGAGACAGTTGGTAGGACACTGGGCAC
