# Supplementary material for: Double-strand breaks in facultative heterochromatin require specific movements and chromatin changes for efficient repair
Source: Nat Commun. 2024 Oct 17;15:8984. doi: 10.1038/s41467-024-53313-2 (PMC11487122; doi:10.1038/s41467-024-53313-2)
Supplement: Supplementary file 3 — Reporting Summary [file 41467_2024_53313_MOESM3_ESM.pdf]

nature portfolio

Corresponding author(s): Aniek Janssen

Last updated by author(s): Sep 9, 2024

Reporting Summary

Nature Portfolio wishes to improve the reproducibility of the work that we publish. This form provides structure for consistency and transparency in reporting. For further information on Nature Portfolio policies, see our Editorial Policies and the Editorial Policy Checklist.

Statistics

For all statistical analyses, confirm that the following items are present in the figure legend, table legend, main text, or Methods section.

n/a	Confirmed	
☐	☒	The exact sample size (*n*) for each experimental group/condition, given as a discrete number and unit of measurement
☐	☒	A statement on whether measurements were taken from distinct samples or whether the same sample was measured repeatedly
☐	☒	The statistical test(s) used AND whether they are one- or two-sided<br>*Only common tests should be described solely by name; describe more complex techniques in the Methods section.*
☒	☐	A description of all covariates tested
☒	☐	A description of any assumptions or corrections, such as tests of normality and adjustment for multiple comparisons
☐	☒	A full description of the statistical parameters including central tendency (e.g. means) or other basic estimates (e.g. regression coefficient) AND variation (e.g. standard deviation) or associated estimates of uncertainty (e.g. confidence intervals)
☐	☒	For null hypothesis testing, the test statistic (e.g. *F*, *t*, *r*) with confidence intervals, effect sizes, degrees of freedom and *P* value noted<br>*Give P values as exact values whenever suitable.*
☒	☐	For Bayesian analysis, information on the choice of priors and Markov chain Monte Carlo settings
☒	☐	For hierarchical and complex designs, identification of the appropriate level for tests and full reporting of outcomes
☒	☐	Estimates of effect sizes (e.g. Cohen's *d*, Pearson's *r*), indicating how they were calculated

Our web collection on statistics for biologists contains articles on many of the points above.

Software and code

Policy information about availability of computer code

Data collection	DeltaVision microscope (DeltaVision Spectris; Applied Precision, LLC) and LD C-Apochromat 63x/1.15 W Korr M27 objective on a LSM880 microscope with Airyscan (Zeiss) were used for image acquisition. Quantitative PCR analyses were performed using Bio-Rad CFX96 Touch Real-Time PCR qPCR system.
Data analysis	Image J was used for image analyses. Graphpad Prism (versions 6 to 9) was used for statistical analyses. Software to analyze repair junctions was described in https://academic.oup.com/nargab/article/4/3/lqac063/6691859. Online tool to analyze repair junctions available on: https://siq.researchlumc.nl/SIQPlotter/

For manuscripts utilizing custom algorithms or software that are central to the research but not yet described in published literature, software must be made available to editors and reviewers. We strongly encourage code deposition in a community repository (e.g. GitHub). See the Nature Portfolio guidelines for submitting code & software for further information.

Data

Policy information about availability of data

All manuscripts must include a data availability statement. This statement should provide the following information, where applicable:

- Accession codes, unique identifiers, or web links for publicly available datasets
- A description of any restrictions on data availability
- For clinical datasets or third party data, please ensure that the statement adheres to our policy

All data generated are included in this article and its supplementary information. The raw targeted sequencing data generated for this study have been deposited in the NCBI SRA database under accession code PRJNA1132713

Research involving human participants, their data, or biological material

Policy information about studies with human participants or human data. See also policy information about sex, gender (identity/presentation), and sexual orientation and race, ethnicity and racism.

Reporting on sex and gender	n.a.
Reporting on race, ethnicity, or other socially relevant groupings	n.a.
Population characteristics	n.a.
Recruitment	n.a.
Ethics oversight	n.a.

Note that full information on the approval of the study protocol must also be provided in the manuscript.

Field-specific reporting

Please select the one below that is the best fit for your research. If you are not sure, read the appropriate sections before making your selection.

☒ Life sciences ☐ Behavioural & social sciences ☐ Ecological, evolutionary & environmental sciences

For a reference copy of the document with all sections, see nature.com/documents/nr-reporting-summary-flat.pdf

Life sciences study design

All studies must disclose on these points even when the disclosure is negative.

Sample size	Samples sizes were chosen in excess to show a significant effect based on pilot experiments used to determine mean and spread.<br>For analyses of microscopy images, a minimum sample size of 20-30 cells per condition was used.<br>For repair pathway analyses using Sanger sequencing and TIDE analyses, a minimum sample size of 5 larvae per condition was used.<br>For ChIP analyses, at least 3 biological replicates were included per condition.
Data exclusions	No data were excluded from this study.
Replication	All experiments have been biologically replicated at least 3 times. The number of replicates has been indicated in all figure legends.
Randomization	No experiments were performed where samples could or should be assigned randomly to a treatment group. Therefore, no group randomization was needed for this study.
Blinding	Investigators were not blinded to group allocation. This was not essential, because the nuclei to be analyzed were selected based on the presence of DNA damage in or outside polycomb domains, which is a similar starting point for all images.

Reporting for specific materials, systems and methods

We require information from authors about some types of materials, experimental systems and methods used in many studies. Here, indicate whether each material, system or method listed is relevant to your study. If you are not sure if a list item applies to your research, read the appropriate section before selecting a response.

Materials & experimental systems

n/a	Involved in the study	
☐	☒	Antibodies
☐	☒	Eukaryotic cell lines
☒	☐	Palaeontology and archaeology
☐	☒	Animals and other organisms
☒	☐	Clinical data
☒	☐	Dual use research of concern
☒	☐	Plants

Methods

n/a	Involved in the study	
☒	☐	ChIP-seq
☒	☐	Flow cytometry
☒	☐	MRI-based neuroimaging

Antibodies

Antibodies used	mouse anti-yH2Av (Developmental Studies Hybridoma Bank, UNC93-5.2.1), 1:250 for immunofluorescence, 5ug for ChIP<br>rabbit anti-H3K27me3 (Invitrogen, MA5-11198), 5ug for ChIP<br>rabbit anti-H2AK119Ub (Cell Signaling, 8240S), 5ug for ChIP<br>rabbit anti-H3K9me3 (Abcam, 176916)., 5ug for ChIP<br>rabbit anti-H3 (Abcam, ab1791), 5ug for ChIP<br>chicken anti-mCherry (abcam, ab205402), 1:1000 dilution for immunofluorescence<br>rabbit anti-Rad51 (gift from Irene Chiolo, USC). 1:500 dilution for immunofluorescence<br>Alexa 488 goat anti-rabbit Invitrogen 1:600 for immunofluorescence (A11029)<br>Alexa 568 goat anti-chicken Invitrogen 1:600 for immunofluorescence (A11041)<br>Alexa 647 goat anti-rabbit Invitrogen 1:600 for immunofluorescence (A21245)
Validation	All antibodies purchased from commercial vendors have validation information on their website.<br>Epicypher SNAP-ChIP K-MetStat panel (19-1001) was used to validate the specificity of the H3K27me3 antibody used for ChIP.<br>yH2Av antibody specificity was determined by observing increased signals using ChIP-qPCR upon single DSB induction in larval tissue.<br>Rad51 antibody specificity was determine by co-localization with yH2Av at later timepoints (>60 min) following DSB induction. The antibody has previously also been extensively validated by the labs of Dr. Kadonaga, Dr. Chiolo and Dr. Karpen.

Eukaryotic cell lines

Policy information about cell lines and Sex and Gender in Research

Cell line source(s)	Kc and S2 wildtype cells were ordered from DGRC: https://dgrc.bio.indiana.edu/Home
Authentication	Kc and S2 lines were authenticated by DGRC upon purchase
Mycoplasma contamination	Cell lines used are negative for mycoplasm (tested 1x per 3 months)
Commonly misidentified lines (See ICLAC register)	No commonly misidentified cell lines were used in this study

Animals and other research organisms

Policy information about studies involving animals; ARRIVE guidelines recommended for reporting animal research, and Sex and Gender in Research

Laboratory animals	DR-white 3eu_1- Janssen laboratory (Janssen et al., 2016)<br>DR-white 3eu_2- generated in this study<br>DR-white 2fH_1- generated in this study<br>DR-white 3fH_1- generated in this study<br>DR-white 3fH_2- generated in this study<br>DR-white 2het_1 - Janssen laboratory (Janssen et al., 2016)<br>DR-white 3het_1 - Janssen laboratory (Janssen et al., 2016)<br>ecDHFR-I-Sce - Janssen laboratory (Janssen et al., 2016)<br>hsp70.HA.I-Sce (hsp.I-Sce) - Jan LaRocque (Do et al., 2014)<br>mu2-eYFP - Janssen laboratory (Janssen et al., 2016)<br>ph-p-mCherry - generated in this study<br>FlyFUCCI - Bloomington #55123<br>UAS:dUtx RNAi - VDRC 105986<br>UAS:ctip RNAi - VDRC 100035<br>UAS:DmKu70 RNAi: BDSC #29594<br>UAS:DmRad51 RNAi: VDRC 13362<br>UAS:luciferase RNAi - Bloomington #31603<br>Act5C-Gal4 - Bloomington #4414 and #3954<br>Da-GAL4 - Bloomington #95282<br>Mei41[29D] - gift from Tin Tin Su<br>dUtx[f01321] - Bloomington #18425

	Age of Drosophila Melanogaster in all experiments: L3 larval stage, except for viability assays in Fig.S8D. In Fig.S8D animals were counted ~1-3 days after eclosion.
Wild animals	n.a.
Reporting on sex	Sex was not considered in this study, because we find it important that the repair effects we see are sex-independent.
Field-collected samples	n.a.
Ethics oversight	Our Drosophila laboratory facilities, access policies, the handling of gmo materials and the training practices for our personnel are in accordance with the guidelines and requirements issued by the Dutch Ministry of Infrastructure and Water Management. We have fully equipped facilities, both at the BSL1 and BSL2 level, including a facility for the handling of (genetically modified) insects

Note that full information on the approval of the study protocol must also be provided in the manuscript.

Plants

Seed stocks	n.a.
Novel plant genotypes	n.a.
Authentication	n.a.
